# Public health and medical care for the world's factory: China's Pearl River Delta Region

**DOI:** 10.1186/1741-7015-9-110

**Published:** 2011-10-04

**Authors:** Guilhem Fabre, Victor G Rodwin

**Affiliations:** 1 Faculty of International Affairs, University of Le Havre, 25 rue Phillipe Lebon, 76057 Cedex, Le Havre, France; 2 Wagner School of Public Service, New York University, Puck Building, 2nd Floor, 295 Lafayette St., New York, NY 10025, USA

## Abstract

While the growth of urbanization, worldwide, has improved the lives of migrants from the hinterland, it also raises health risks related to population density, concentrated poverty and the transmission of infectious disease. Will megacity regions evolve into socially infected breeding grounds for the rapid transmission of disease, or can they become critical spatial entities for the protection and promotion of population health? We address this question for the Pearl River Delta Region (PRD) based on recent data from Chinese sources, and on the experience of how New York, Greater London, Tokyo and Paris have grappled with the challenges of protecting population health and providing their populations with access to health care services. In some respects, there are some important lessons from comparative experience for PRD, notably the importance of covering the entire population for health care services and targeting special programs for those at highest risk for disease. In other respects, PRD's growth rate and sheer scale make it a unique megacity region that already faces new challenges and will require new solutions.

## Introduction

The city is both a center for disease and poor health and also a place of hope, cures and good health. From the earliest times, the city has attracted the poor and been the target of plague, as well as war. Likewise, the health care industry has always been part of the economic base of cities -- from Lourdes, in France, to Rochester, Minnesota, to megacities (defined by the United Nations Development Program as urban agglomerations with a population exceeding ten million) and their surrounding regions. With its highly disproportionate share of health resources, including hospitals, physicians, nurses and social services, megacity regions around the world include centers of excellence in health and social care. Yet, as Richard Horton, editor of *The Lancet*, once noted, '... for all of its rational efficiency and benevolent intent, the city is likely to be the death of us' [1].

Will megacity regions evolve into socially infected breeding grounds for the rapid transmission of disease? Or can they become critical spatial entities for the protection and promotion of population health? We address this question based on recent data from Chinese sources, and on the experience of how New York, Greater London, Tokyo and Paris have grappled with the challenges of protecting population health and providing their populations with access to health care services.

## China's Pearl River Delta Region (PRD) in Guangdong Province

China's PRD lies in the central southern coastal part of Guangdong, a southern province adjacent to Hong Kong, which includes nine municipalities each with well over a million inhabitants (Table 1). With a population of more than 47 million, nearly half of Guangdong Province, spread out over almost a quarter of Guangdong's land mass, PRD accounts for more than 80% of its gross domestic product (GDP) [2,3]. The official 2007 population figures noted in Table 1 underestimate the present population and may not account fully for the immense migrant population. According to more recent reports from the *Yangcheng wanbao (Yangcheng Evening News)*, Shenzhen had a total population of 14.5 million, 12 million as floating population and 2.5 million permanent residents [4].

**Table 1:** Population Estimates (in millions) for Nine Principal Cities of Pearl River Delta Region

Guangzhou	10.1
Shenzhen	8.6
Dongguan	6.9
Foshan	5.9
Zhaoqing	3.8
Huizhou	3.9
Jiangmen	4.1
Zhongshan	2.5
Zhuhai	1.5
Total	47.3

*Source: Guangdong *tongji nianjian *(Guangdong statistical yearbook)*, 2008, p.528 [16].

PRD's concentration of economic activity is also apparent at the national level. With only 3.6% of China's population on 0.6% of its land mass, PRD accounts for 10% of China's GDP, nearly 30% of the cumulative stock of foreign direct investment (FDI) and the same proportion of the nation's exports. PRD is, in earnest, the heart of China, as the world's factory, for its export-led growth, largely outsourced from Hong Kong, has focused on textiles, toys, a full range of consumer goods and electronic products (computers, Televisions, i-Pods and i-Phones) that are shipped around the world.

The PRD's growth was fueled from two sources: 1) massive amounts of external capital, originally from Hong Kong and later from the rest of the world; and 2) a massive inflow of 20 million unskilled migrant laborers from rural villages, which allowed China to maintain its competitive advantage in labor-intensive activities. Over the period between 1978 to 2007, PRD's GDP grew at an annual rate of 21.2%, more than twice the national average [5]. It is now the wealthiest city-region of China (outside of Beijing and Shanghai), and a strategic hub for China's national economic development plans.

The State Council produced a PRD plan for the period 2008 to 2020, which focuses largely on infrastructure, such as highways, rail and water transport for containerized shipping, and now a massive project to erect a bridge connecting the mainland, Hong Kong and Macao. The plan calls for continued growth (3,000 km) of highway construction between 2008 and 2020. In addition, between 2012 and 2020, it projects massive growth of rail construction for high-speed trains (from 1,100 to 2,200 km), augmented capacity for ports along the coast, and increased airport capacity for the region (from 80 to 150 million passengers) [6]. Behind these specific investments looms the idea of improved integration among PRD's nine cities thereby creating the largest megacity of the world. Recent news, however, suggests that a more feasible approach is likely to promote integration across three groups of cities: Guangzhou-Foshan-Zhaoqing in the northwest, Jiangmen-Zhongshan-Zhuhai on the west bank of the Pearl River, and Shenzhen-Dongguan-Huizhou on the east bank [7].

Given these planning efforts for PRD, one might expect more attention to have been paid to human capital requirements for continued growth, particularly following the brutal slowdown due to the global economic crisis of 2008-2009, when unemployment surged, millions of migrants returned to their villages, and economists pondered future development models for the region beyond export-led growth [5]. Indeed, the State Council Plan for PRD invokes the need for investments in a range of public health initiatives and health care resources but success in this area is more difficult to achieve than laying down roads and tracks and increasing port and airline capacity. What is to be done about millions of migrants who suffer harsh working conditions in a region with unprecedented levels of income inequality, population density, pollution, and stress, all of which present enormous challenges for infrastructure development, workplace regulation, environmental protection and public health?

## Public health and access to health care

Migrants have contributed massively to PRD's industrialization but they have also grown to include an impoverished population with pressing social problems. In cities such as Shenzhen and Dongguan, the ratio of migrants officially registered with these cities to those born there is, respectively, six and four to one. The social problems that accompany this scale of migration, such as high numbers of vagrants, many with severe and often contagious diseases, and abandoned children, have led Provincial authorities to hire 40,000 social workers to manage family planning services, temporary housing, and other social services [8].

Beyond the problems of migrants who have not found employment, conditions for the employed are notoriously harsh and industrial accidents are rampant. The most immediate public health challenge in PRD arises from working conditions of migrant laborers who take on all the jobs that local citizens refuse, for example employment in mines, quarries, construction and petrochemical plants which are the sectors with the highest rates of accidents and occupational health problems. Many companies employ migrants in the most dangerous jobs, expose them to dangerous substances, and skimp on safety measures [8,9].

Even in assembly-line production, enforcement of occupational health and safety standards are lax and hours of work are dangerous to human health. One survey, for example, reports that 63.8% of migrants work seven days a week without rest, and that the average work week (for 63.9% of them) is 56 hours [10]. This situation reflects the exceedingly low salary structure for migrant labor. Before the strikes of 2010, the basic wage was around 900 Yuan (less than $140) a month thus forcing laborers to work overtime to earn enough to live and save a small margin. The spate of last year's suicides at the Foxconn electronics plant in Shenzhen [11] (the biggest factory in the world with 300,000 employees) are directly linked to these conditions.

## Challenges that must be faced

PRD faces a stupendous challenge with regard to public health and access to basic health services because these issues are dealt with separately by each city prefecture. There are flagrant inequalities in coverage between urban and rural areas [12-14]. Consider the case of Shenzhen, PRD's next largest city after Guangzhou and the municipal government reputed to be most aggressive in confronting the broader health and social problems of the region. Here, in addition to high rates of industrial accidents, the local government confronts a high incidence of infectious diseases (including AIDS, drug-resistant tuberculosis, and malaria), rising chronic disease burdens, a high prevalence of mental problems, and maternal and children's health issues [15].

Guangdong Province is characterized by striking inequalities in access to health care. Although there will, no doubt, be improved access for the poor after the recent health care reforms in China are implemented, analysis of official Chinese statistics from 1985 to 2008, when most of the poor were responsible for paying for health care themselves (out-of-pocket), indicate that health care inequalities were considerably greater in Guangdong Province than in the nation as a whole. The ratio of per capita health care expenditure of the highest income to the lowest income groups, as measured in the Chinese census, was one to six in Guangdong in contrast to a nation-wide ratio of 2.78 [16,17]. When health care is funded out of public funds, such ratios are typically less than one because the poor are sicker than the more well-to-do and spend more time in hospitals due to the fact that they usually receive less medical attention before their conditions deteriorate.

Within PRD, such inequalities are more pronounced because of the disparities between urban and rural areas, in income and insurance coverage, and the continued exclusion of most migrant laborers from social insurance ranging from accident, maternity, pensions and basic health care coverage. As noted by Peng Guifang, in 2009, less than 10% of migrants have basic social security coverage and 80% have no health insurance coverage [8]. Although this is supposed to change as the new national health insurance legislation is implemented, it looms as an enormous challenge for local experts who have already attributed the labor shortages of 2004 and 2007 to inadequate social insurance cover [10].

In addition to providing health care coverage to a growing population, it will be necessary for PRD planners to strengthen the region's public health infrastructure, that is, the capacity of local officials to perform the core functions of public health: 1) assessment through the systematic collection, analysis, and dissemination of information on the health of the community; 2) health policy development based on analysis of available evidence of health risks and effective responses; and 3) assurance to constituents that necessary services are available [18]. The capacity of local officials to perform these functions will depend, in part, on the size and quality of their workforce; their information systems for epidemiological surveillance; and the organizational links they can forge to implement regulations and deliver public health services.

## Lessons from other megacity regions

Experience from other megacity regions in wealthy nations, notably New York, London, Paris and Tokyo, is important because they have survived devastating disease epidemics in the past and have established a strong public health infrastructure. All four cities are characterized by significant disparities in income, educational attainment, unemployment rates, housing and environmental conditions among their neighborhoods. These social determinants of health have important implications for how to target health protection and promotion programs and for how to improve emergency preparedness and communication with diverse urban populations. In New York, London and Paris, public health leaders must target programs for immigrant populations from around the world. In PRD, the challenge is to target such programs to migrant populations from within China.

Despite their achievements in improving population health over the past century, these wealthy megacity regions still confront onerous health risks: 1) the return of infectious diseases (for example drug-resistant tuberculosis) and the emergence of new ones, such as AIDS, severe acute respiratory syndrome (SARS), and the avian flu; 2) terrorism, including bio-terrorism, and emergencies stemming from climate changes, for example, heat waves; 3) barriers in access to medical services for ethnic minorities and/or the poor; and 4) rising inequalities among social groups. In response to these problems, they have developed programs for those who fall through the cracks and sophisticated surveillance systems to monitor disease outbreaks. The development and management of such public health infrastructure involves important links between local, regional, national as well as private non-profit organizations representing civil society and global health authorities. It is likely that in PRD greater efforts will be required to strengthen local, regional and private non-profit organizations.

New York City stands out, in contrast to London, Paris and Tokyo, because it has the largest share of its population not covered under a national system that eliminates financial barriers to health care access. In contrast, it excels with respect to its disease surveillance systems because it has one of the most sophisticated ones, particularly with respect to syndromic surveillance [19]. Beyond these differences, and the contrasts with respect to public health organization, there are convergent trends in public health interventions. Among all four cities, there is increasing awareness, among public health leaders, that the neighborhood is a critical spatial unit for targeted interventions for those populations at highest risk of disease [20]. Since megacity regions are characterized by spatial disparities in population and neighborhood characteristics this response is not surprising.

## Inventing the future

In contrast to the quantitative targets and efforts to improve integration of physical infrastructure linking many of PRD's cities, the State Council's Development Plan for PRD has produced no more than a catalogue of propositions for desirable health care investments and reforms. Comparative experience, however, as well as an important assessment by Li Wenhui, emphasize the importance of regional integration for organizing public health priorities, emergency care services, unified social insurance systems across municipal governments, and universal coverage across diverse occupational groups, urban and rural populations, as well as migrants [21,22].

In Li Wenhui's view, universal health insurance coverage is a necessary condition for improving the capacity of the public health system to respond to health threats. Indeed, without reliable clinical information on all those who fall ill, it is impossible to organize effective disease surveillance and prevention programs that meet critical public health challenges. Yet, as we have noted, some of the most successful megacities have found that universal coverage, although necessary, is not sufficient for building effective public health infrastructure. It is also critical to identify high-risk groups and neighborhoods, and to organize targeted programs for them. Finally, in tackling these problems, it is critical to allow diverse groups representing civil society to obtain funding from government authorities and global nongovernmental organizations.

In some respects, there are some important lessons for PRD from comparative experience. In other respects, its growth rate and sheer scale make it a unique megacity region that already faces new challenges and will require new solutions. First, pollution levels are dangerous. The economic loss caused by adverse health effects from air pollution due to particulate matter alone is estimated to be between 0.75% and 1.35% of PRD's GDP [23]. Second, problems related to additives used by the food processing industry remain rampant despite a national campaign to control their use [24,25]. Third, the risks posed by what Laurie Garret calls 'microbial hitch-hikers,' are especially high. PRD, as the world's factory, is not only the largest area of internal migration within China; it is also the largest gateway city-region in the world with respect to numbers of foreigners entering the nation. Finally, over the long-run, PRD is no more immune than other coastal cities to climate change and its effects on sea levels, temperature rises and their potential catastrophic effects on human populations, particularly older people. What makes this more troublesome in PRD are the potential effects of such small changes on the vast scale of the region's population and economic activity [26,27].

## Summary and conclusions

PRD faces formidable challenges in public health and overcoming barriers in access to health care: 1) a massive influx of migrants, many with associated social problems; 2) harsh working conditions and associated industrial accidents; 3) a high incidence of infectious diseases (including AIDS, drug-resistant tuberculosis, and malaria), rising chronic disease, a high prevalence of mental problems and maternal and children's health issues; 4) inequalities in income and access to health care; 5) pollution, dangerous food additives and risks related to the large number of foreigners entering China through PRD; and 6) possible long-run effects of climate change on the coastal region.

To meet these challenges and become a megacity region that succeeds in protecting its population from disease and promoting health, PRD planners would do well to study the experience of other megacity regions in wealthy nations because they share similar challenges and have demonstrated areas of success. The unique characteristics of PRD, however, suggest that prevailing models will not suffice. PRD planners will have to go beyond learning from others; they will have to innovate and come up with new solutions as they invent the future.

## Abbreviations

GDP: gross domestic product; FDI: foreign direct investment; PRD: Pearl River Delta Region; SARS: severe acute respiratory syndrome.

## Competing interests

The authors declare that they have no competing interests.

## Authors' contributions

The paper was written jointly by both authors. GF was responsible for the translation and analysis of Chinese source material, based on previous work on the Chinese socio-economy. VR brought the health policy perspective based on previous work on health care in world cities. Both authors read and approved the final manuscript.

## Authors' information

Guilhem Fabre is professor of Chinese Civilization and Economy at the Faculty of International Affairs, University of Le Havre. He is the author of *Criminal Prosperity: Drug Trafficking, Money Laundering and Financial Crises after the Cold War*, RoutledgeCurzon, 2003 and of *Propriété intellectuelle, contrefaçon et innovation: Les multinationales face à l'économie de la connaissance*, Publication des universités de Rouen et du Havre, 2009. Victor Rodwin is professor of Health Policy and Management, Wagner School of Public Service, New York University and author (with Michael Gusmano and Daniel Weisz) of *Health Care in World Cities: New York, London, Paris*. Baltimore, Johns Hopkins University Press, 2010; and *Growing Older in World Cities: New York, London, Paris and Tokyo *(with Michael Gusmano). Vanderbilt U. Press, 2006.

## Pre-publication history

The pre-publication history for this paper can be accessed here:

http://www.biomedcentral.com/1741-7015/9/110/prepub
